# Supplementary material for: Prediction of Human Inhibition Brain Function with Inter-Subject and Intra-Subject Variability
Source: Brain Sci. 2020 Oct 13;10(10):726. doi: 10.3390/brainsci10100726 (PMC7600619; doi:10.3390/brainsci10100726)
Supplement: Supplementary file 1 [file brainsci-10-00726-s001.pdf]

Supplementary Material

Prediction of Human Inhibition Brain Function with Inter-Subject and Intra-Subject Variability

Rupesh Kumar Chikara, Li-Wei Ko*

* Correspondence: Li-Wei Ko (lwko@mail.nctu.edu.tw)

1.1. Power Spectral Density (PSD) of Intra-Subject Variability during Response Inhibition

The **Figure 1** shows the single subject-1 intra-subject variability in power spectral density (PSD) at brain regions related to inhibition included frontal lobe (F3, F4), motor cortex (C3, C4), parietal lobe (P3, P4) and occipital lobe (O1, O2) under successful stop and failed stop trials. The EEG signals were segmented after the onset of the stimulus from 1ms to 500ms to observe the PSD of EEG signals under successful stop and failed stop conditions. Asterisk show significant difference between successful stop trials and failed stop trials by Wilcoxon signed rank test (*$p < 0.05$). The delta (1-4Hz) and theta (4-7Hz) band powers were slightly increased in successful stop trials than in failed stop trials at F3, F4 (frontal lobe). However, we found the delta (1-4Hz) and theta (4-7Hz) band powers were slightly decreased in successful stop trials than in failed stop trials at C3, C4 (motor cortex), P3, P4 (parietal lobe) and O1, O2 (occipital lobe).

In addition, **Figure 1** displays the single subject-2 intra-subject variability in PSD over F3, F4, C3, C4, P3, P4, O1 and O2 channels. We examined the delta (1-4Hz), theta (4-7Hz) and alpha (8-12Hz) band powers were slightly increased in failed stop trials than in successful stop trials in F3, F4, C3, C4, P3, P4, O1 and O2 channels. The intra-subject variability in PSD of subject-3 to subject-20 is presented in **Figure 2** to **Figure 10**.

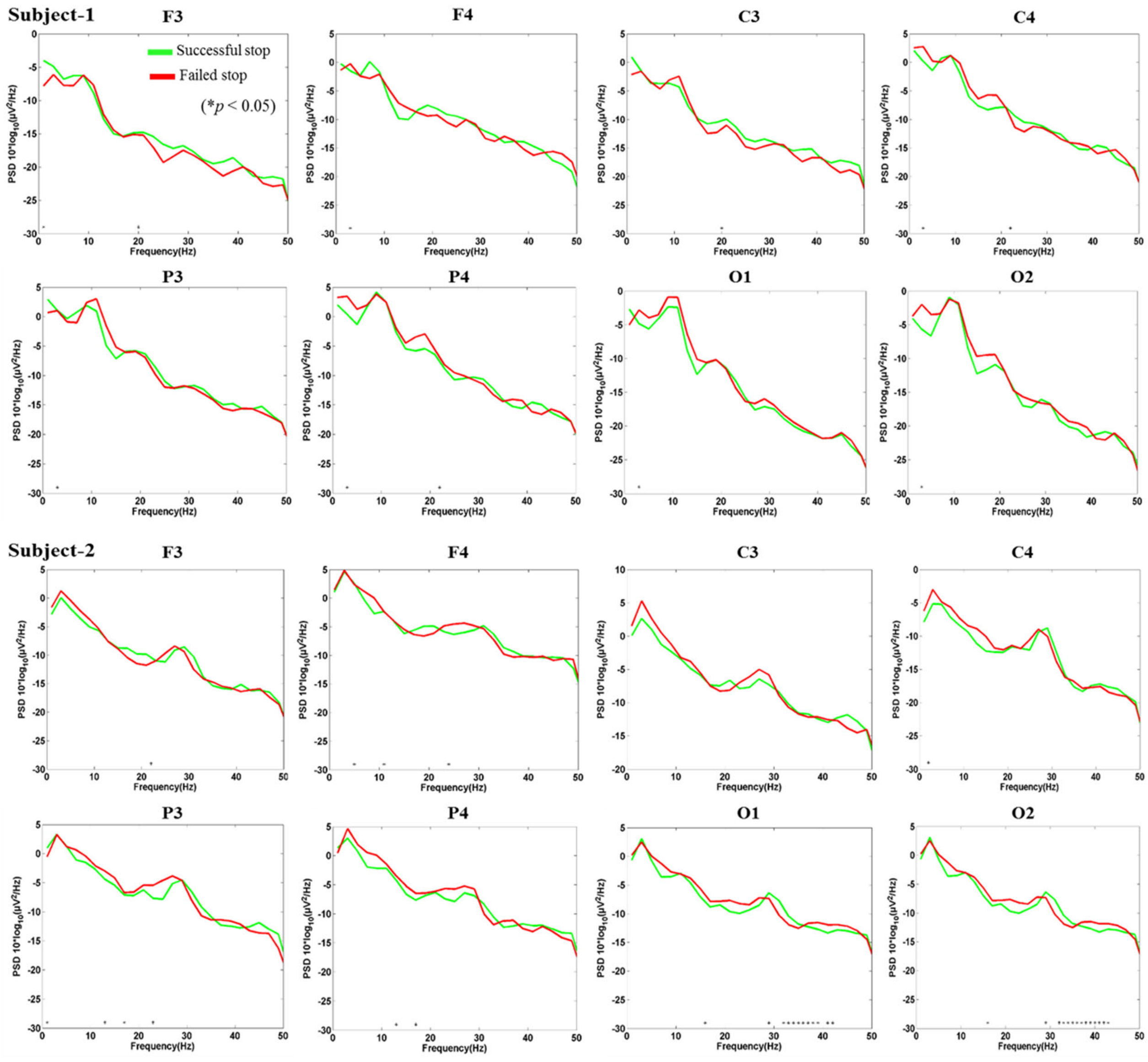

Supplementary Figure 1. The EEG signals power spectral density (PSD) of single subject-1 and subject-2 (intra-subject variability) at F3, F4, C3, C4, P3, P4, O1 and O2 under successful stop and failed stop trials. Asterisk show significant difference between successful stop trials and failed stop trials by Wilcoxon signed rank test at (*$p < 0.05$).

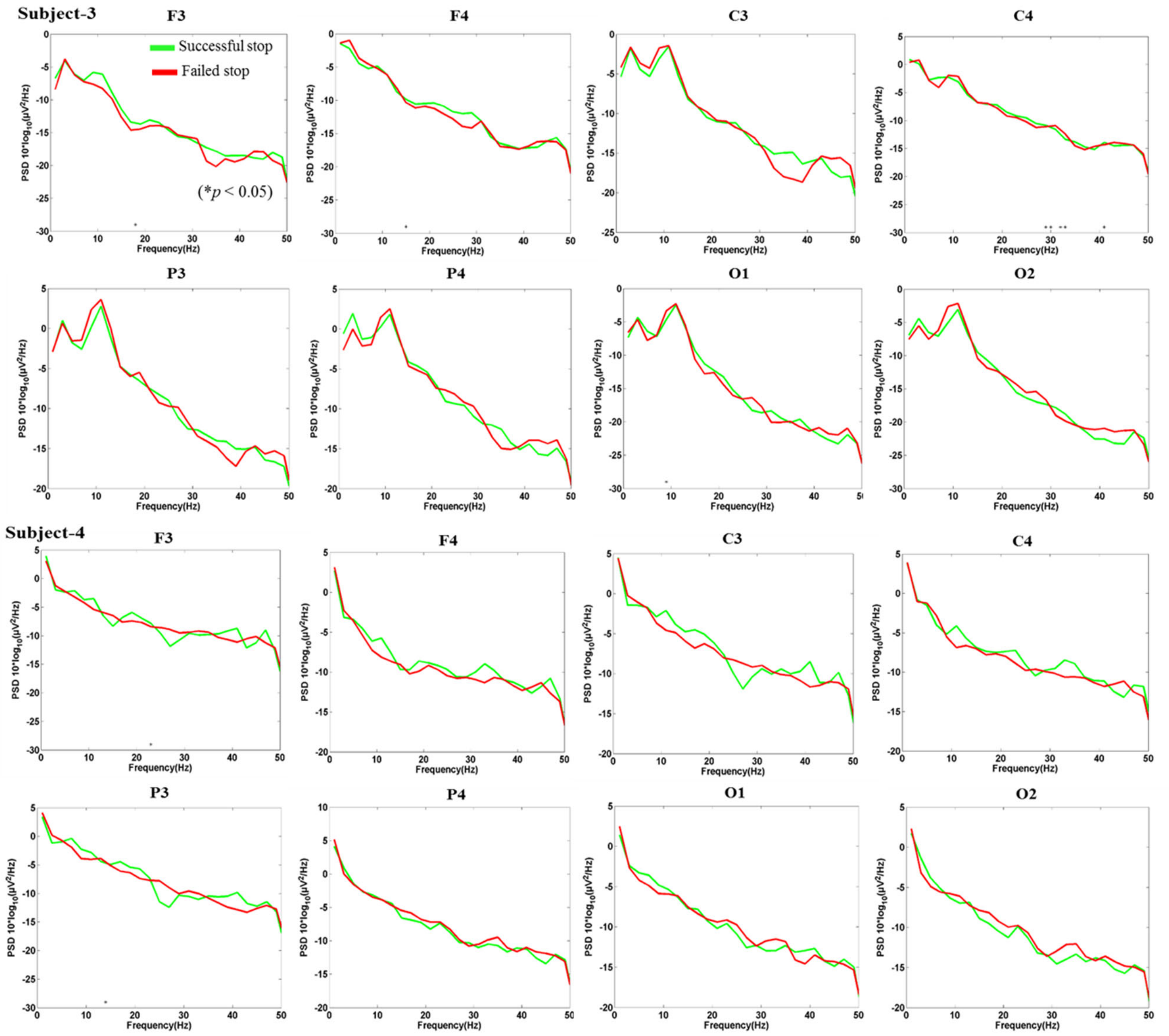

Supplementary Figure 2. The EEG signals PSD of subject-3 and subject-4 (intra-subject variability) at F3, F4, C3, C4, P3, P4, O1, and O2 under successful stop and failed stop trials. Asterisk show significant difference between successful stop trials and failed stop trials by Wilcoxon signed rank test (*$p < 0.05$).

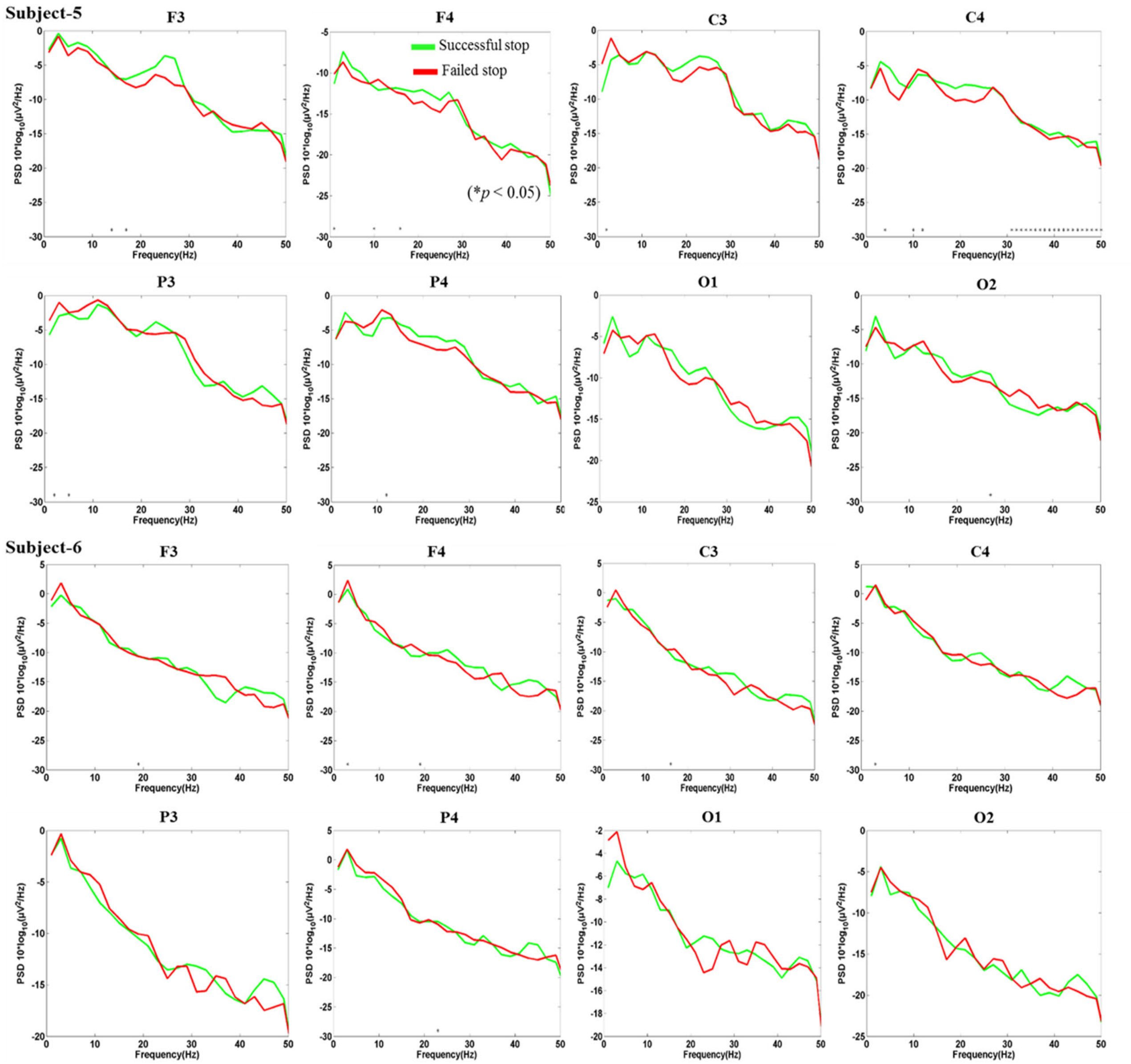

Supplementary Figure 3. The EEG signals PSD of subject-5 and subject-6 (intra-subject variability) at F3, F4, C3, C4, P3, P4, O1, and O2 under successful stop and failed stop trials. Asterisk show significant difference between successful stop trials and failed stop trials by Wilcoxon signed rank test (*$p < 0.05$).

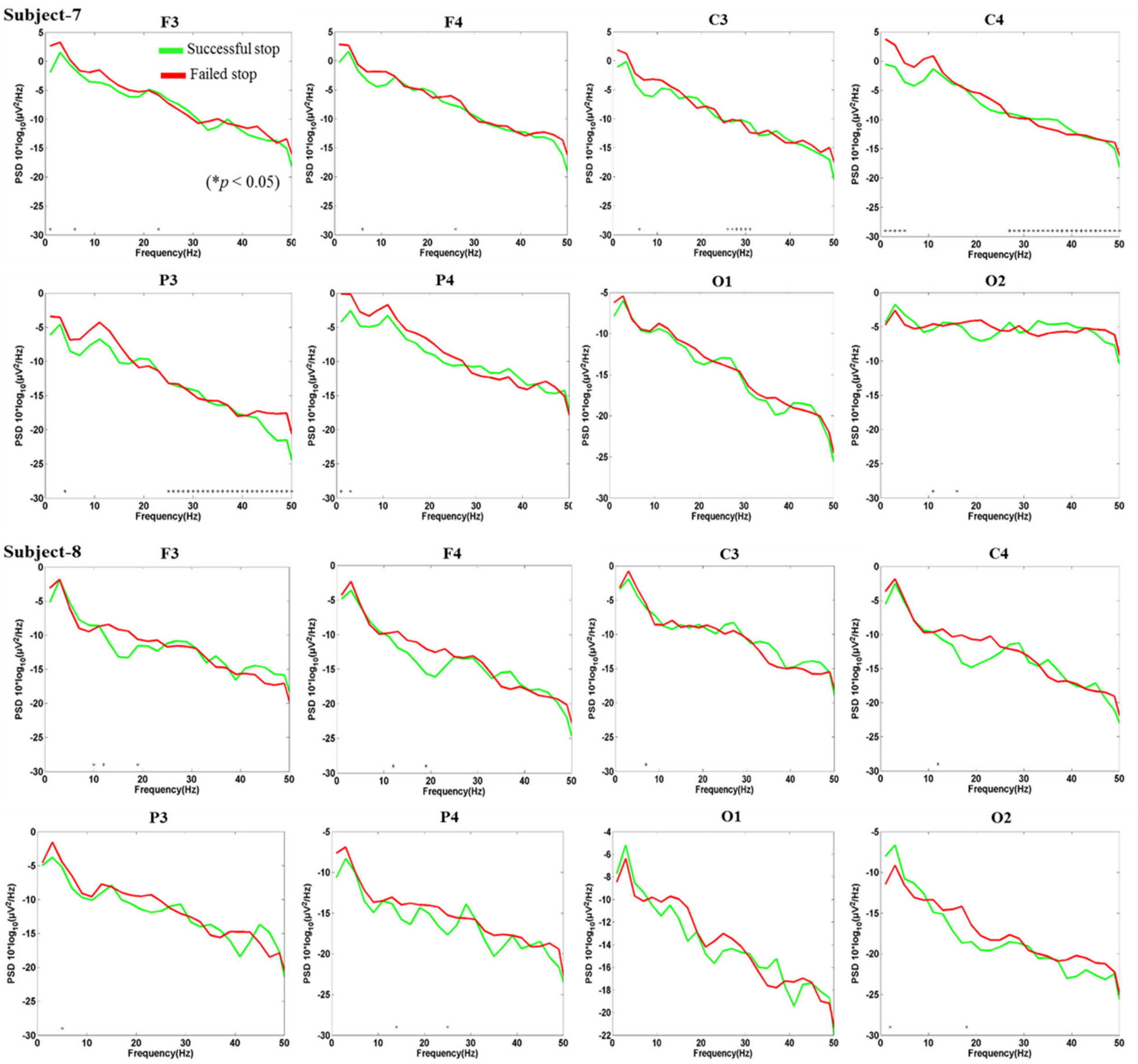

Supplementary Figure 4. The EEG signals PSD of subject-7 and subject-8 (intra-subject variability) at F3, F4, C3, C4, P3, P4, O1, and O2 under successful stop and failed stop trials. Asterisk show significant difference between successful stop trials and failed stop trials by Wilcoxon signed rank test (*$p < 0.05$).

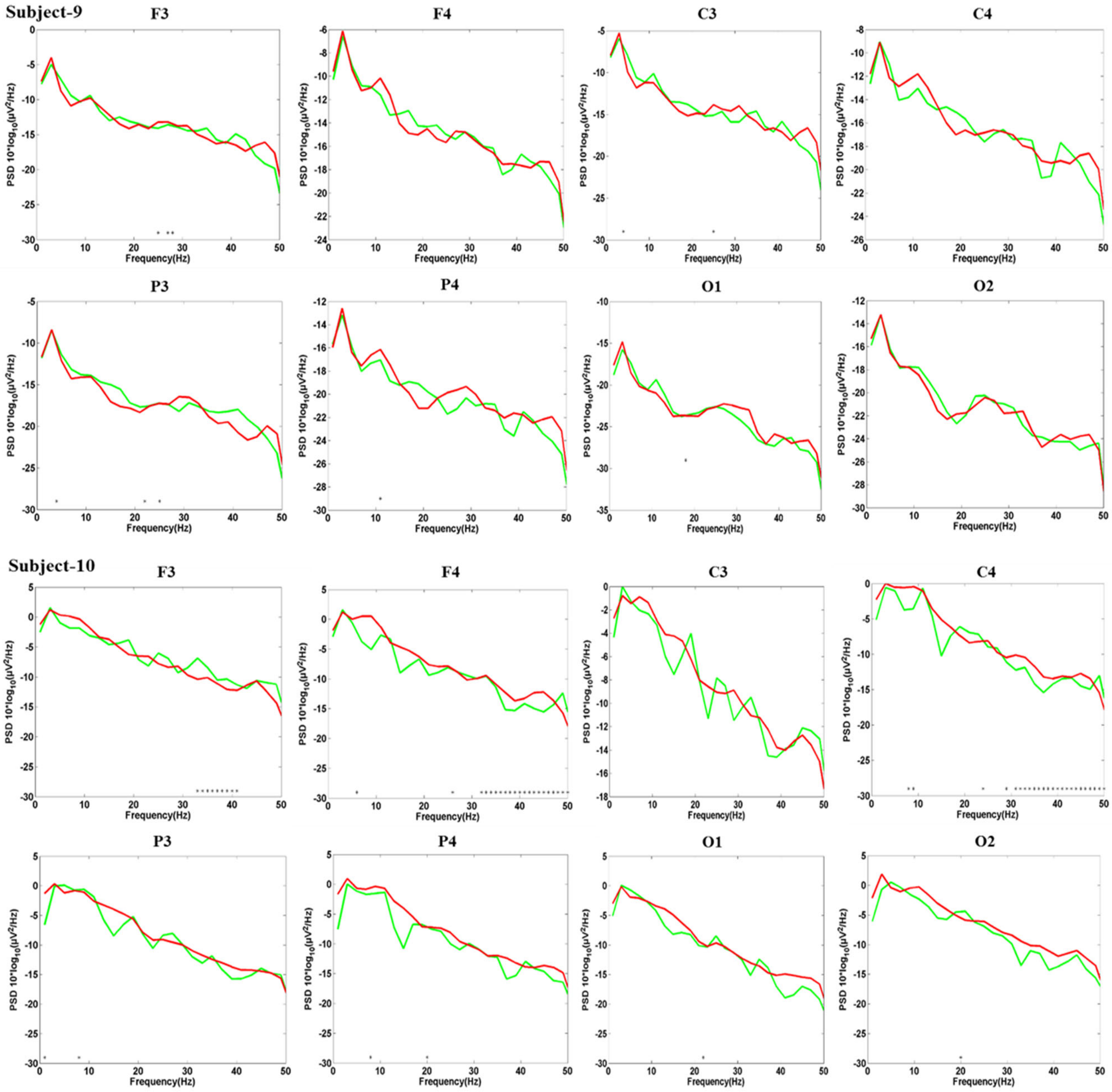

Supplementary Figure 5. The EEG signals PSD of subject-9 and subject-10 (intra-subject variability) at F3, F4, C3, C4, P3, P4, O1, and O2 under successful stop and failed stop trials. Asterisk show significant difference between successful stop trials and failed stop trials by Wilcoxon signed rank test (*$p < 0.05$).

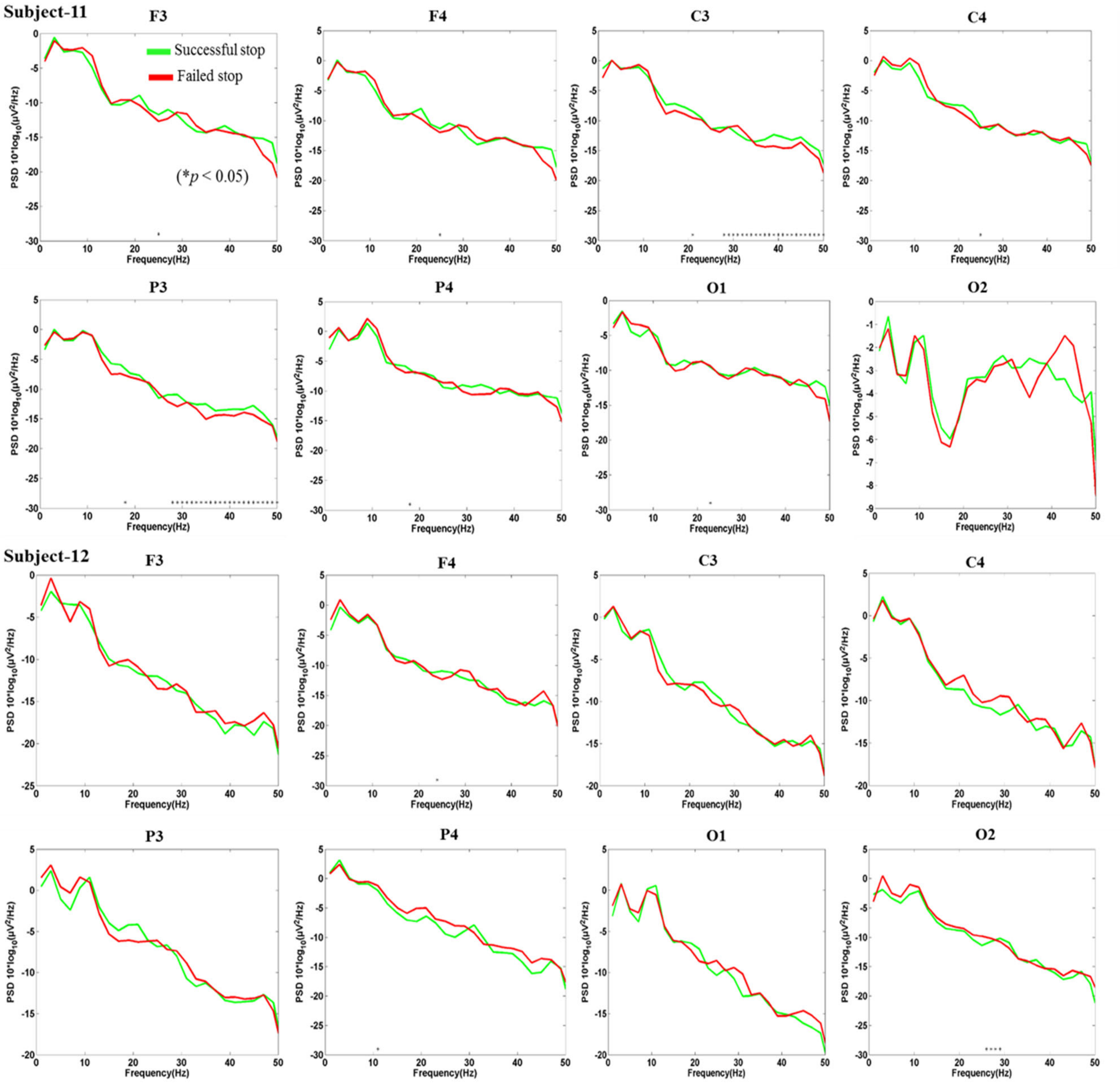

Supplementary Figure 6. The EEG signals PSD of subject-11 and subject-12 (intra-subject variability) at F3, F4, C3, C4, P3, P4, O1, and O2 under successful stop and failed stop trials. Asterisk show significant difference between successful stop trials and failed stop trials by Wilcoxon signed rank test (*$p < 0.05$).

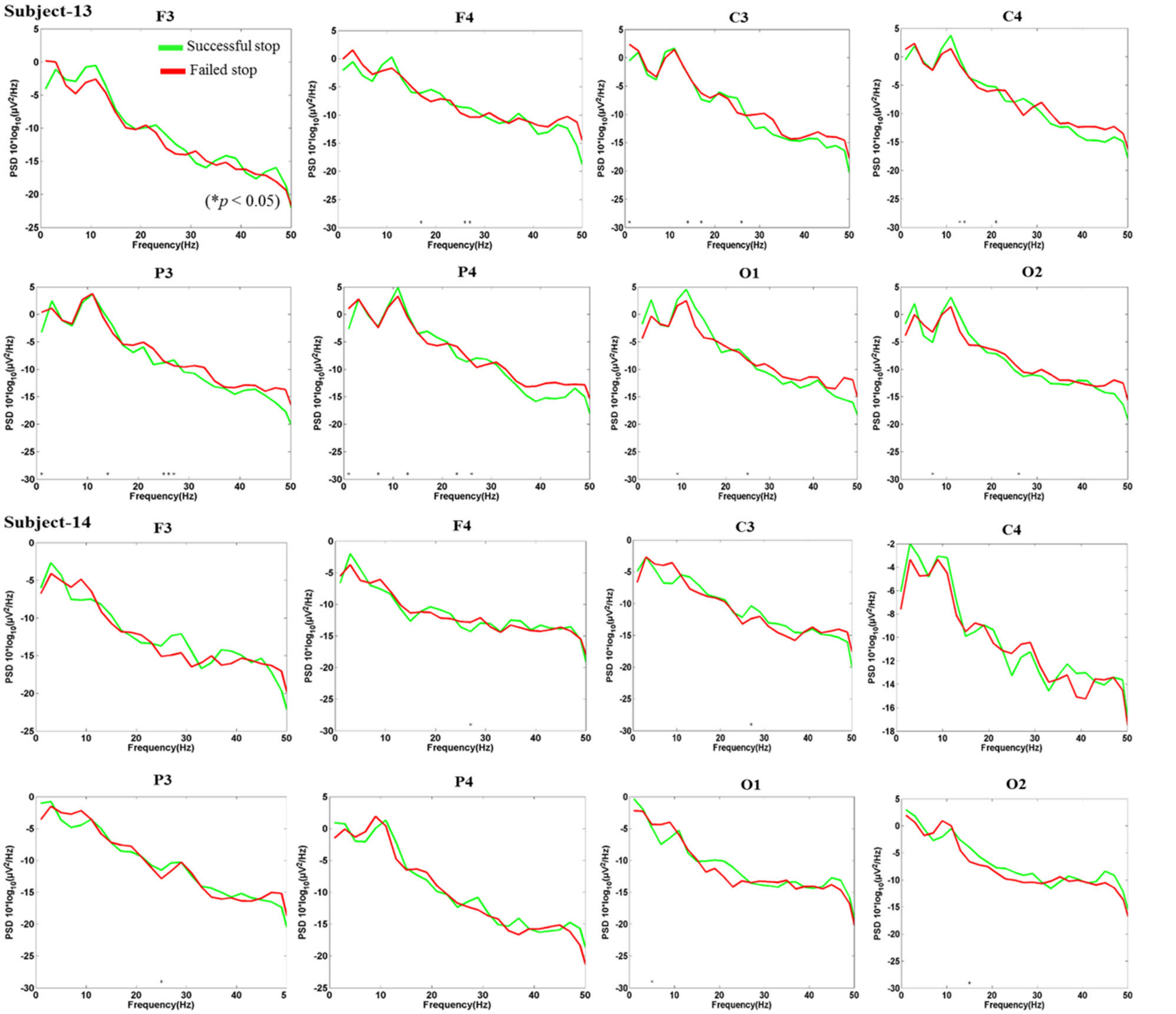

Supplementary Figure 7. The EEG signals PSD of subject-13 and subject-14 (intra-subject variability) at F3, F4, C3, C4, P3, P4, O1, and O2 under successful stop and failed stop trials. Asterisk show significant difference between successful stop trials and failed stop trials by Wilcoxon signed rank test (*$p < 0.05$).

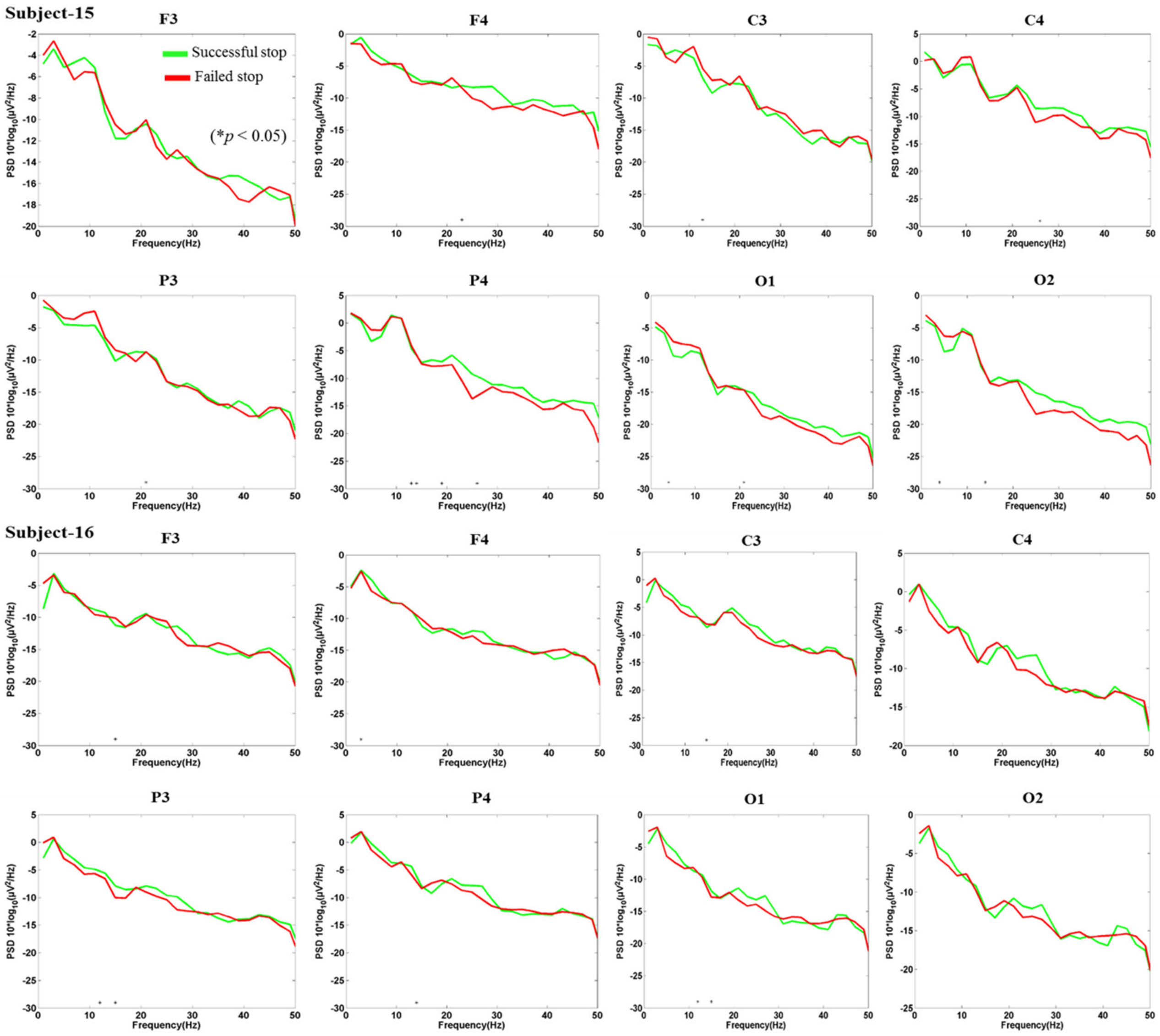

Supplementary Figure 8. The EEG signals PSD of subject-15 and subject-16 (intra-subject variability) at F3, F4, C3, C4, P3, P4, O1, and O2 under successful stop and failed stop trials. Asterisk show significant difference between successful stop trials and failed stop trials by Wilcoxon signed rank test (*$p < 0.05$).

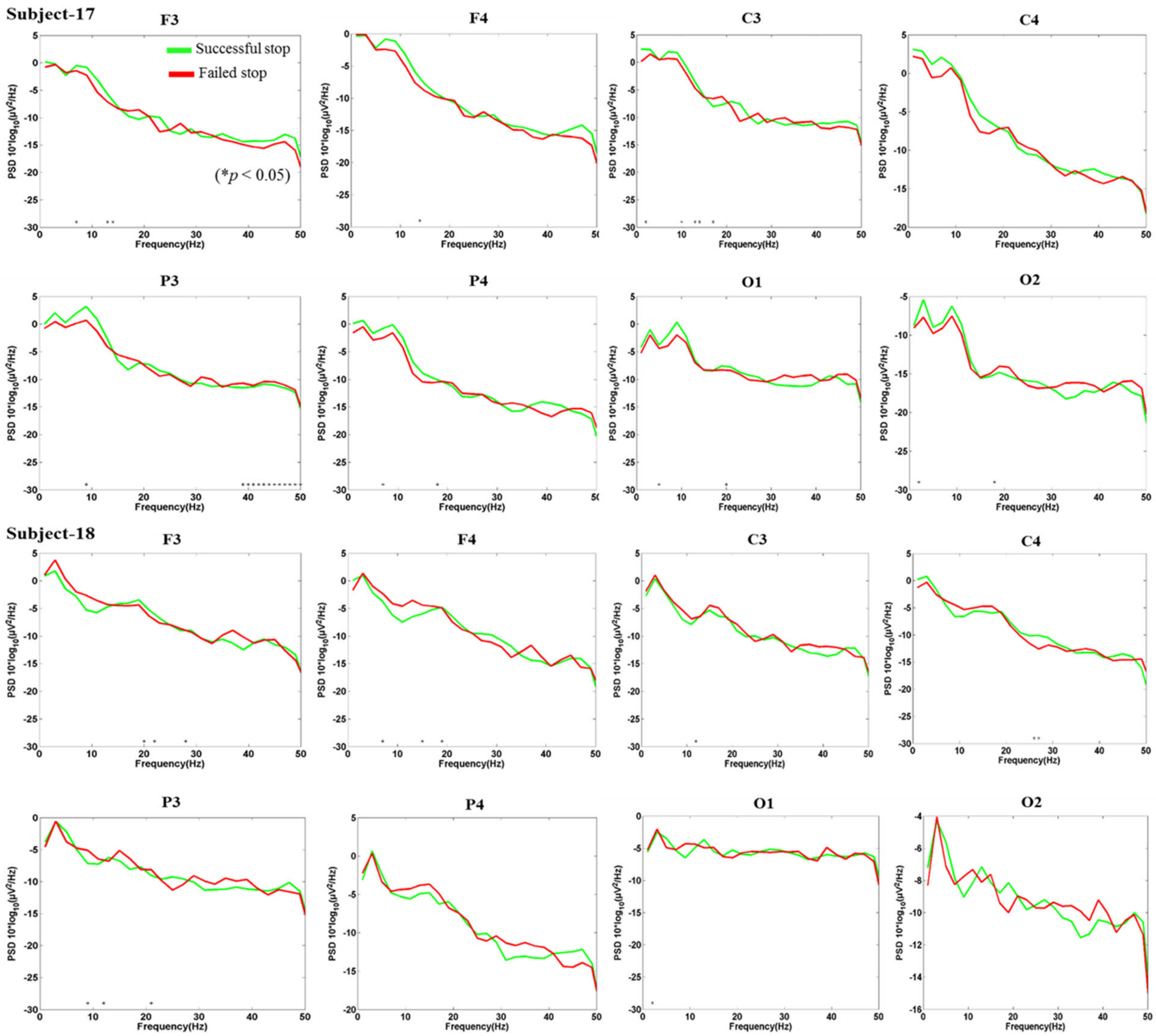

Supplementary Figure 9. The EEG signals PSD of subject-17 and subject-18 (intra-subject variability) at F3, F4, C3, C4, P3, P4, O1, and O2 under successful stop and failed stop trials. Asterisk show significant difference between successful stop trials and failed stop trials by Wilcoxon signed rank test (*$p < 0.05$).

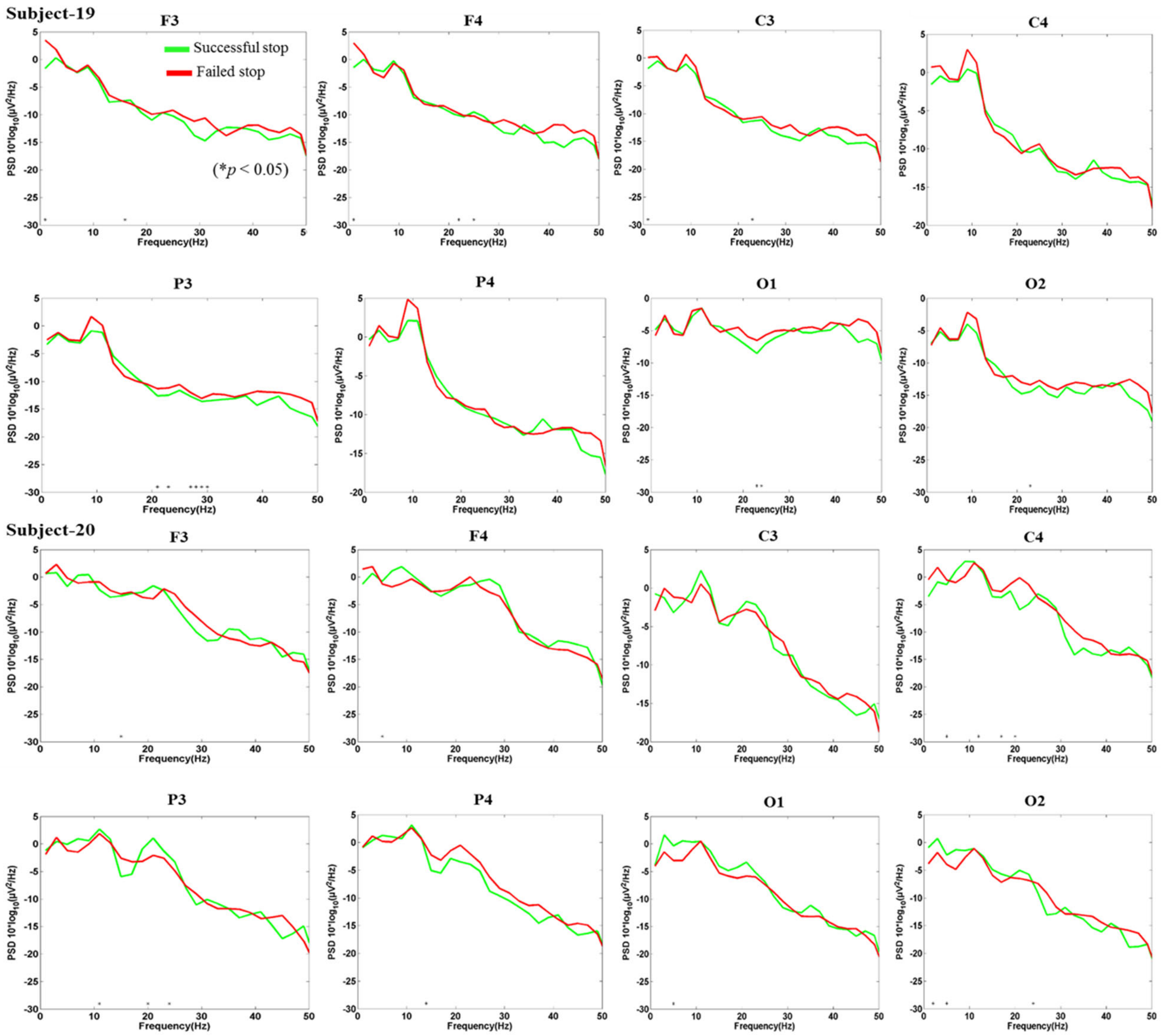

Supplementary Figure 10. The EEG signals PSD of subject-19 and subject-20 (intra-subject variability) at F3, F4, C3, C4, P3, P4, O1, and O2 under successful stop and failed stop trials. Asterisk show significant difference between successful stop trials and failed stop trials by Wilcoxon signed rank test (*$p < 0.05$).
